# Modulation of Rat Cancer-Induced Bone Pain is Independent of Spinal Microglia Activity

**DOI:** 10.3390/cancers12102740

**Published:** 2020-09-24

**Authors:** Marta Diaz-delCastillo, Rie Bager Hansen, Camilla Kristine Appel, Lykke Nielsen, Sascha Nolsøe Nielsen, Konstantinos Karyniotakis, Louise M. Dahl, Rikke B. Andreasen, Anne-Marie Heegaard

**Affiliations:** Department of Drug Design and Pharmacology, University of Copenhagen, DK-2100 Copenhagen, Denmark; rikkerie.hansen@sund.ku.dk (R.B.H.); camilla.k.appel@gmail.com (C.K.A.); lykkenielsen93@gmail.com (L.N.); saschanolsoe@hotmail.com (S.N.N.); k.karyniotakis@gmail.com (K.K.); louisedahl92@gmail.com (L.M.D.); rbandreasen@hotmail.com (R.B.A.); amhe@sund.ku.dk (A.-M.H.)

**Keywords:** cancer-induced bone pain, microglia, spinal cord, animal models

## Abstract

**Simple Summary:**

Cancer-induced bone pain is one of the most debilitating and feared symptoms of cancer patients. Many patients have inadequate pain relief with the current treatment options, and there is a need for new pain medication targeting the mechanisms of cancer-induced bone pain. It has been hypothesized that microglia (cells involved in central nervous system homeostasis) are involved in the signalling of cancer-induced bone pain; however, data from animal models are inconsistent. Here, we apply immunohistochemical staining of the microglial markers ionizing calcium-binding adaptor molecule 1 (Iba-1) and phosphorylated p38-mitogen-activated protein kinase (P-p38 MAPK) to show that microglial reaction is not a feature of cancer-induced bone pain; this independently of disease stage, animal sex or cancer cell-line. Furthermore, pharmacological inhibition of microglia did not affect pain-related behaviours in cancer-bearing rats. Overall, our data support that microglial reaction is not a main player in cancer-induced bone pain.

**Abstract:**

The dissemination of cancer to bone can cause significant cancer-induced bone pain (CIBP), severely impairing the patient’s quality of life. Several rodent models have been developed to explore the nociceptive mechanisms of CIBP, including intratibial inoculation of breast carcinoma cells in syngeneic Sprague Dawley rats. Using this model, we investigated whether resident spinal microglial cells are involved in the transmission and modulation of CIBP, a long-debated disease feature. Immunohistochemical staining of ionizing calcium-binding adaptor molecule 1 (Iba-1) and phosphorylated p38-mitogen-activated protein kinase (P-p38 MAPK) showed no spinal microglial reaction in cancer-bearing rats, independently of disease stage, sex, or carcinoma cell line. As a positive control, significant upregulation of both Iba-1 and P-p38 was observed in a rat model of neuropathic pain. Additionally, intrathecal administration of the microglial inhibitor minocycline did not ameliorate pain-like behaviors in cancer-bearing rats, in contrast to spinal morphine administration. Our results indicate that microglial reaction is not a main player in CIBP, adding to the debate that even within the same models of CIBP, significant variations are seen in disease features considered potential drug targets. We suggest that this heterogeneity may reflect the clinical landscape, underscoring the need for understanding the translational value of CIBP models.

## 1. Introduction

The dissemination of cancer to bone can lead to significant pain, with up to 85% of patients experiencing cancer-induced bone pain (CIBP) [1,2,3]. The patient with CIBP typically presents with a somewhat steady background pain and, as the disease progresses, breakthrough pain episodes, which are transient, severe pain exacerbations [4,5,6]. That said, the patient’s pain experiences are heterogeneous and there may be differences in the underlying pathology requiring different treatment strategies [7,8].

CIBP is particularly difficult to relieve with the available analgesic therapies [9,10], and there is an apparent need for novel therapeutic approaches to handle both background and breakthrough pain. Drug development is guided by the promise of targets, but, as many analgesic drug trials fail [11], it may be time to reconsider whether the models developed for CIBP are exploited in the best possible way when translating the results into the clinic [12,13,14].

To better understand the mechanisms underlying CIBP, several rat and mouse models have been developed over the last three decades. The most commonly used involve the inoculation of syngeneic carcinoma cell lines in the intramedullary cavity of a long bone (e.g., tibia or femur), leading to the localized development of osteolytic bone lesions and pain-like behaviors [15,16,17]. These models have been useful to advance our knowledge of CIBP, demonstrating that this pain type shares nociceptive and neuropathic pain components but also presents with its own characteristics and neurochemical signature [18,19,20].

Microglia are a diverse population of resident macrophage-like cells in the central nervous system (CNS) that play a crucial role in tissue homeostasis. First described in 1975 [21], their role in pain has become increasingly studied. Under physiological conditions, microglia survey the neuronal tissue with their ramified processes and play a crucial role in maintaining CNS health by engulfing and pruning superfluous synapses and eliminating dead cells, protein aggregates, and other damaging residues in the brain and spinal cord [22]. Upon nerve injury, microglial cells become reactive and hypertrophic and their number in the dorsal horn of the spinal cord increases, a process termed microgliosis [23]. Reactive microglia release interleukins and other factors that, in turn, stimulate immune cells and spinal neurons, ultimately contributing to the transmission of the pathological nociceptive signal [24]. While microglial involvement in rodent models of neuropathic pain is well established [23,25], it remains controversial whether microglial reaction is a main feature of CIBP and, in essence, whether this is a drug target that should be pursued.

Here, we investigated whether microglial reaction plays a central role in pain transmission in the commonly used Walker 256 breast cancer model of CIBP. Through immunohistochemical characterization of ionizing calcium-binding adaptor molecule 1 (Iba-1) and phosphorylated p38-mitogen-activated protein kinase (P-p38 MAPK), two markers of microglial reaction [25], and through pharmacological microglial inhibition our findings suggest that microglial reaction is not a main feature of this model of CIBP; this is independently of disease stage, rat sex, and cancer cell line inoculated. However, instead of dismissing microglia as a possible target, we suggest that these diverse features can be exploited to reflect the heterogeneity of the patient population. Thus, further characterization of the models is crucial to understand their potential translational validity.

## 2. Results

### 2.1. Intratibial Inoculation of Walker 256 Cancer Cells Causes Cancer-Induced Bone Pain in Female Rats

**Figure 1:**
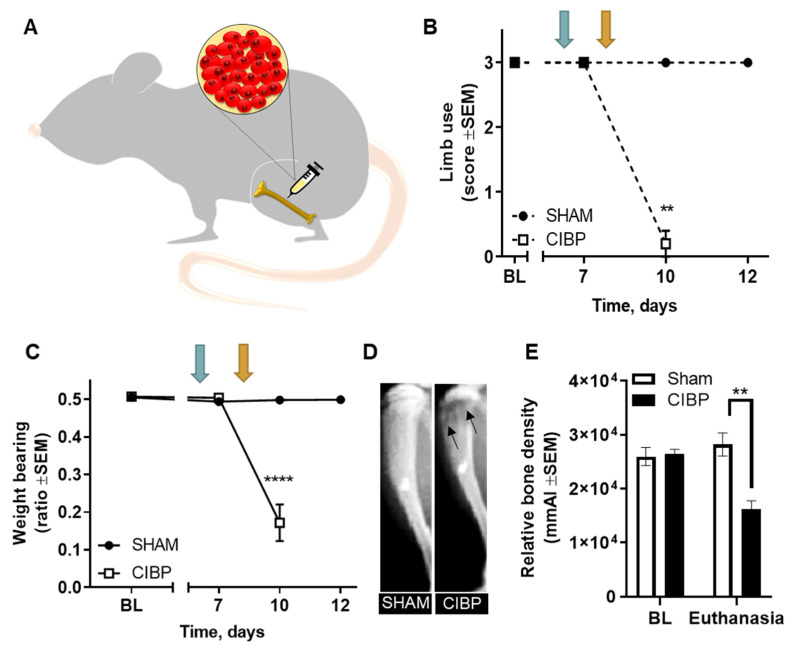
The Walker 256 rat model of cancer-induced bone pain (CIBP). (**A**) A rat model of CIBP was set up through intratibial inoculation of 10^5^ Walker 256 breast carcinoma cells in female Sprague Dawley rats. (**B**,**C**) Cancer-bearing rats presented significantly lower limb-use scores (**B**) and weight-bearing ratios (**C**) at post-surgical day 10, compared with sham. (**D**) Representative X-ray images displaying a sham tibia (left) and a cancer-bearing tibia (right) and endpoint. Arrows indicate areas of bone degradation. (**E**) Intratibial cancer cell inoculation led to a significantly lower relative bone density in cancer-bearing tibias compared with baseline; no changes in relative bone density were observed in sham tibias. (**B**,**C**). Blue arrows indicate the time point just before the development of pain-like behaviors (post-surgical day 6) and orange arrows illustrate the early stage of CIBP, where the pain-like behaviors initiate (post-surgical day 8). Sham n = 5; CIBP n = 5. BL = baseline. Data are presented as mean ± standard error of the mean (SEM). ** *p* < 0.01; **** *p* < 0.0001.

The Walker 256 rat model was used to investigate whether microglia are involved in the nociceptive transmission of CIBP (Figure 1A). Cancer-bearing rats displayed pain-related behaviors, measured as a decrease in limb-use score and weight-bearing ratio at post-surgical day 10 (*F*_1,8_ = 196.00 *p* < 0.01 for limb use; *F*_2,16_ = 43.52, *p* < 0.0001 for weight bearing; n = 5; Figure 1B,C). Moreover, cancer cell inoculation led to the development of osteolytic bone lesions, measured as a significant decrease in the relative bone density of cancer-bearing tibias, compared with sham (*F*_1,8_ = 19.73, *p* < 0.01; n = 5; Figure 1D).

### 2.2. Absence of Microglial Reaction in the Ipsilateral Dorsal Horn of Female Rats Inoculated with Walker 256 Cancer Cells

Temporal microglial reaction has been described to be involved in different phases of CIBP, either at the early or late stages of disease progression; however, the findings are ambiguous, with reports of microglial reaction being important for the initiation of CIBP but not its maintenace, and vice versa [26,27]. Thus, we examined whether microglial reaction is present just before the development of measureable nociception (post-surgical day 6), in the initial phase of the nociceptive phenotype (post-surgical day 8), and in the progressed stage of CIBP (defined as the humane end point, at limb-use score 0).

**Figure 2:**
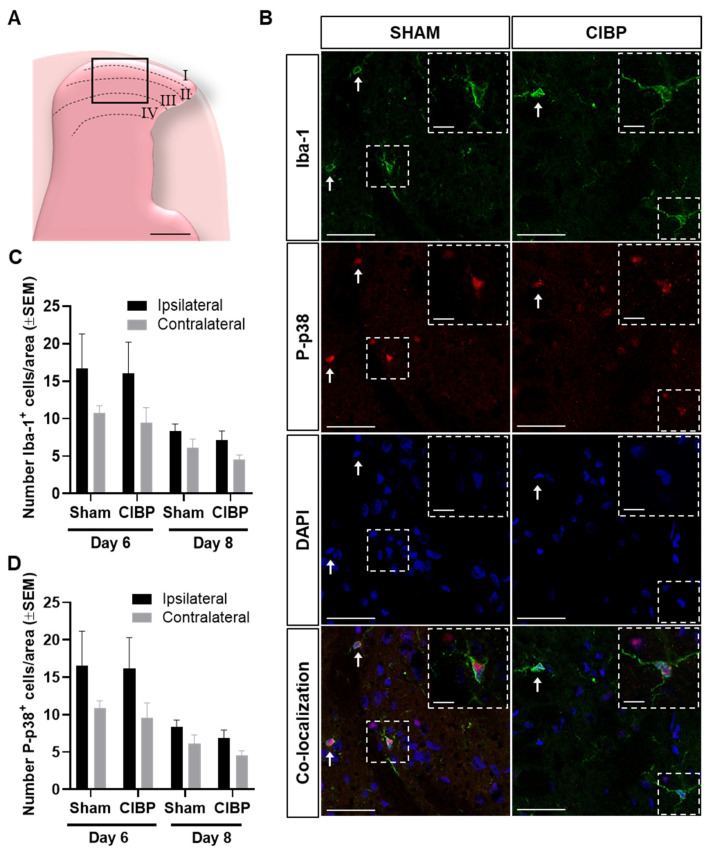
Immunohistochemical characterization of microglial reaction in the female Walker 256 model of CIBP. (**A**) Diagram demonstrating the area of the spinal cord dorsal horn used for analysis (347 × 260 µm^2^). Scale bar indicates 250 µm. (**B**) Representative confocal images of microglia characterization (Iba-1, P-p38, DAPI, and co-localization) in the lumbar spinal cord of sham and cancer-bearing rats. Arrows indicate Iba-1^+^, P-p38^+^, and DAPI^+^ cells. Arrows indicate examples of microglia cells expressing Iba-1^+^, P-p38, and DAPI^+^. Images were obtained with a 40× objective lens, scale bars indicate 50 µm, and scale bars in the inserts indicate 10 µm. (**C**) The number of Iba-1^+^ cells was not significantly higher in the ipsilateral dorsal horn of the spinal cord of cancer-bearing rats, compared with the contralateral side and with sham rats on post-surgical days 6 or 8. (**D**) The number of P-p38^+^ cells did not significantly differ between the ipsilateral and contralateral dorsal horns, nor between the ipsilateral dorsal horns of cancer-bearing and sham rats on post-surgical days 6 or 8. For each time point: Sham n = 5, CIBP n = 6. Data are presented as mean ± SEM.

**Figure 3:**
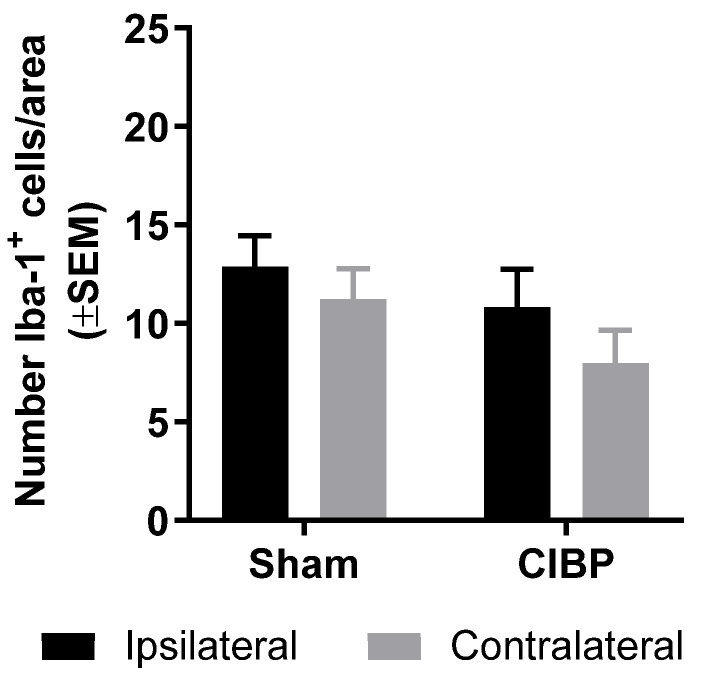
Advanced CIBP in females did not induce spinal microglial reaction. The number of Iba-1^+^ cells was not significantly higher in the ipsilateral dorsal horn of the spinal cord of female cancer-bearing rats, compared with sham, at the humane endpoint (limb-use score of 0). The number of Iba-1^+^ cells was not significantly different between the ipsilateral and contralateral dorsal horns of cancer-bearing or sham rats. Sham n = 5, CIBP n = 5. Data are presented as mean ± SEM.

Immunohistological analyses of the dorsal horn of the spinal cord revealed a lack of significant differences in the numbers of Iba-1^+^ or P-p38^+^ cells between the ipsilateral and contralateral dorsal horn of cancer-bearing or sham rats, as well as between the ipsilateral dorsal horns of cancer-bearing and sham rats, both at post-surgical days 6 and 8 (day 6: *F*_1,18_ = 3.57, *p* > 0.05; day 8: *F*_1,18_ = 5.54, *p* > 0.05; n = 5–6; Figure 2B,C). Interestingly, a trend toward increased Iba-1 and P-p38 cells was observed on post-surgical day 6, but not on day 8; this trend toward microglial reaction could be related to post-surgical nociception [28]. In the progressed stage of CIBP, no significant difference was found in the number of spinal Iba-1^+^ cells between the ipsilateral and contralateral dorsal horn of cancer-bearing or sham rats. Likewise, no significant difference was found in the number of Iba-1^+^ cells in the ipsilateral dorsal horn of cancer-bearing rats, compared with sham (*F*_1,16_ = 2.45, *p* > 0.05; n = 5, Figure 3), demonstrating that even in the late stages of CIBP, we did not detect microglial reaction in female rats innoculated with Walker 256 cancer cells.

### 2.3. Absence of Microglial Reaction Is Independent of Cancer Cell Line Inoculated

**Figure 4:**
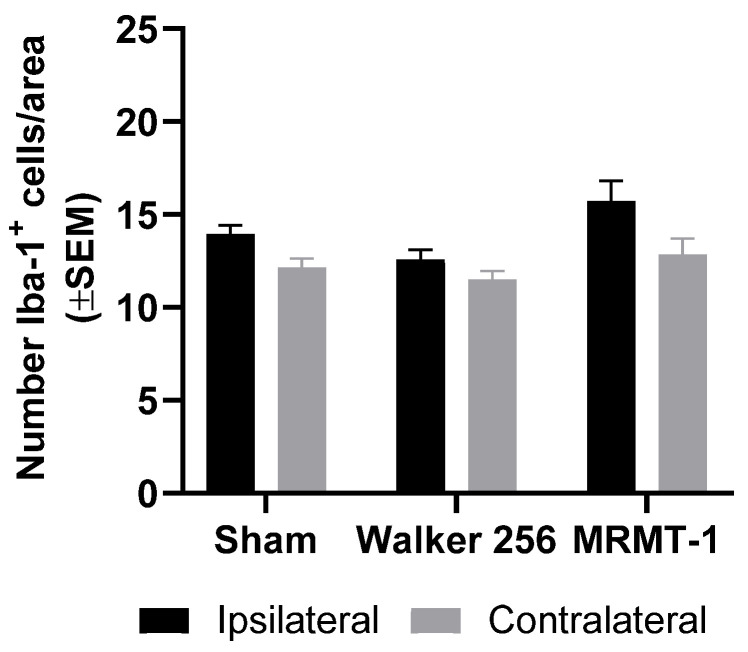
CIBP does not induced microglial reaction in two different CIBP rat models. Intratibial inoculation of MRMT-1 or Walker 256 carcinoma cells did not cause a significant upregulation of Iba-1^+^ cells in the ipsilateral dorsal horn of the spinal cord of cancer-bearing rats compared with sham. Sham n = 10; Walker 256 n = 7; MRMT-1 n = 6. Data are presented as mean ± SEM.

To evaluate whether the observed lack of microglial reaction in this CIBP rat model was exclusive to the inoculation of Walker 256 breast carcinoma cells, independent experiments were performed where Sprague Dawley rats were inoculated with the syngeneic MRMT-1 breast carcinoma cell line and compared to Sprague Dawley rats inoculated with the Walker 256 cell line. No significant differences were observed in the numbers of Iba-1^+^ cells in the ipsilateral dorsal horn in either group of cancer-bearing rats compared with the ipsilateral horn of sham rats (*F*_2,40_ = 5.23, *p* > 0.05; n = 6–10; Figure 4). Also, neither of the models showed a significant increase in the number of Iba-1^+^ cells in the ipsilateral dorsal horn compared with the contralateral dorsal horn (*F*_1,40_ = 13.12, *p* > 0.05; n = 6–10; Figure 4). Both Walker 256- and MRMT-1-inoculated rats developed significant pain-related behaviors and osteolytic lesions over time, confirming disease phenotype development (Supplementary Materials Figure S1).

### 2.4. Absence of Microglial Reaction in Male Rats Inoculated with Walker 256 Cancer Cells

**Figure 5:**
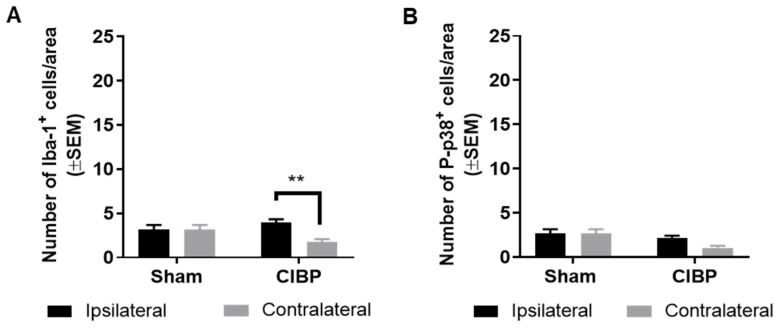
CIBP did not induce microglial reaction in male rats. (**A**) No significant difference was found in the number of spinal Iba-1^+^ cells between the ipsilateral dorsal horn of cancer-bearing and sham rats (*p* > 0.05). Cancer-bearing rats showed a significant increase in the number of Iba-1^+^ cells in the ipsilateral dorsal horn of the spinal cord compared with the contralateral dorsal horn. (**B**) No significant differences were detected in the numbers of P-p38^+^ cells within or among groups. Sham n = 6, CIBP n = 8. ** *p* < 0.01. Data are presented as mean ± SEM.

Sexual dimorphism in microglial reaction has previously been described in rodent models of chronic neuropathic pain [27,29]. While most studies investigating the role of spinal microglia in the Walker 256 model of CIBP focus on females [26,30,31,32], we also addressed whether microglial reaction was present in cancer-bearing male rats. No significant difference was found in the number of Iba-1^+^ cells between the ipsilateral dorsal horn of cancer-bearing and sham rats (*F*_1,24_ = 0.55, *p* > 0.05; n = 6–8; Figure 5A), indicating a lack of microglial reaction. A small but significant increase of Iba-1+ cells was found in the ipsilateral dorsal horn of cancer-inoculated rats when compared to the contralateral site (*F*_1,24_ = 6.86, *p* < 0.01; n = 6–8; Figure 5A). The lack of microglial reaction was further supported by the absence of significant differences in P-p38 expression among groups, demonstrating that microglial reaction is also absent in the male Walker 256 model of CIBP (*F*_1,24_ = 8.95, *p* > 0.05; n = 6–8; Figure 5B). As expected, innoculation of Walker 256 cancer cells in male Sprague Dawely rats led to the development of pain-like behaviors over time, as well as a decrease in relative bone density (Figure S2).

### 2.5. Microglial Reaction Is Observed in the Spinal Cord of Rats with Spared Nerve Injury

**Figure 6:**
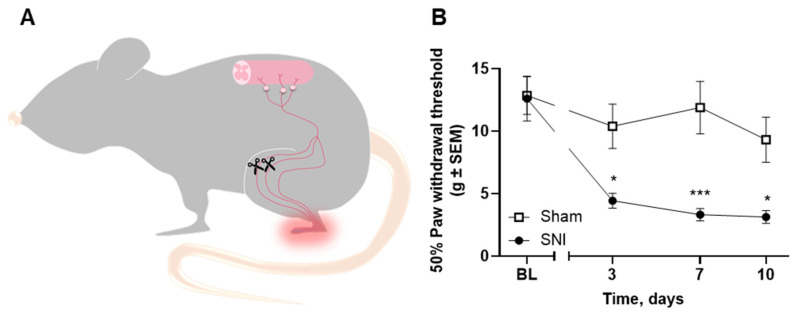
The spared nerve injury (SNI) rat model. (**A**) The SNI rat model of neuropathic pain was established through surgical sectioning of the common peroneal and tibial branches of the sciatic nerve. (**B**) SNI-operated rats presented a significantly lower mechanical threshold from post-surgical day 3. Sham n = 6, SNI n = 7. Data are presented as mean ± SEM. * *p* < 0.05; *** *p* < 0.001.

To demonstrate the robustness of our method to detect Iba-1 and P-p38 upregulation, a rat model of neuropathic pain was established. Male rats underwent the spared nerve injury (SNI; Figure 6A) where the common peroneal and tibial branches of the sciatic nerve are sectioned. This leads to the development of mechanical hyperalgesia on the lateral side of the operated hind paw. In the von Frey test, the SNI-operated rats showed a significantly lower paw withdrawal threshold from post-surgical day 3 when compared with sham rats (*F*_3,33_ = 5.83, *p* < 0.05 on days 3 and 10, *p* < 0.001 on day 7; n = 6–7; Figure 6B).

**Figure 7:**
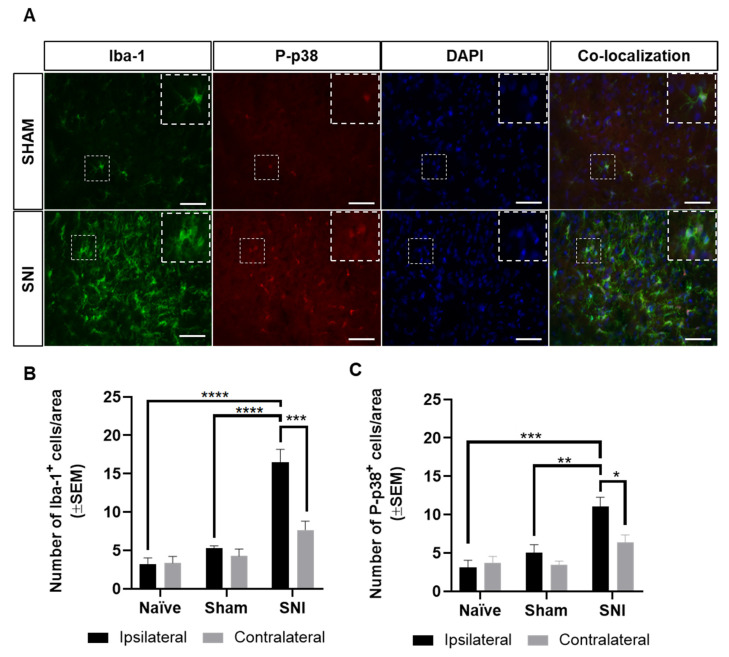
Immunohistochemical characterization of microglial reaction in the SNI model. (**A**) Representative confocal microscopy images of microglia characterization (Iba-1 and P-p38) in the lumbar spinal cord of sham and SNI-operated rats. Co-localization of Iba-1 and P-p38 was estimated as 97.7% (n = 3). Note that the microglia depictured in the insert of SNI-operated rats demonstrate two P-p38+ and Iba-1+ microglia and a microglia only positive for Iba-1. Images were obtained with a 40× objective lense, and scale bars indicate 50 µm. (**B**) The ipsilateral dorsal horn of SNI-operated rats presented a significantly higher number of Iba-1^+^ cells compared with contralateral side, and with the ipsilateral dorsal horn of naïve and sham rats. (**C**) The ipsilateral dorsal horn of the lumbar spinal cord of SNI-operated rats presented a significant upregulation in the number of P-p38^+^ cells compared with the contralateral side and with the ipsilateral dorsal horn of naïve and sham rats. (**B**,**C**) Naïve n = 3, sham n = 4, SNI n = 5. Data are presented as mean ± SEM. * *p* < 0.05; ** *p* < 0.01; *** *p* < 0.001; **** *p* < 0.0001.

Lumbar (L4) spinal sections from sham and SNI-operated rats were visualized by confocal microscopy to confirm microglia morphology and co-localization of P-p38 with microglia cells. Microglia in sham rats appeared with fine, ramified processes, while microglia in SNI-operated rats had the more amoboid appearance characteristic of microglia reaction (Figure 7A). On average, 97.7% of P-p38^+^ cells were found to also express Iba-1 in the the ipsilateral dorsal horn of SNI-opereated rats (range of P-p38^+^ and DAPI^+^ cells counted: 45–86, range of P-p38^+^, Iba-1^+^, and DAPI^+^ cells counted 44–83, n = 3).

As SNI male rats presented pronounced mechanical hyperalgesia at post-surgical day 7, the spinal cords of male naïve, sham, and SNI Sprague Dawley rats were collected 7 days after surgery for immunohistological characterization of the expression of Iba-1 and P-p38. As expected, SNI rats showed significant microglial reaction in the ipsilateral dorsal horn compared to the ipsilateral dorsal horn of both sham and naïve rats (*F*_2,18_ = 33.79, Iba-1: *p* < 0.0001, P-p38: *p* < 0.01; Iba-1: *p* < 0.0001, P-p38: *p* < 0.001, respectively; n = 3–5; Figure 7A–C). Moreover, a significant upregulation of Iba-1^+^ and P-p38^+^ cells was found in the ipsilateral dorsal horn of the SNI-operated rats compared with the contralateral dorsal horn (*F*_1,18_ = 11.02, Iba-1: *p* < 0.001, P-p38: *p* < 0.05; n = 5; Figure 7A–C). No significant differences were found between the ipsilateral and contralateral dorsal horn of naïve or sham rats or between the ipsilateral side of naïve and sham rats (n = 3–5; Figure 7A–C).

### 2.6. Pharmacological Inhibition of Spinal Microglia Does not Ameliorate Nociception in the CIBP Rat Model

**Figure 8:**
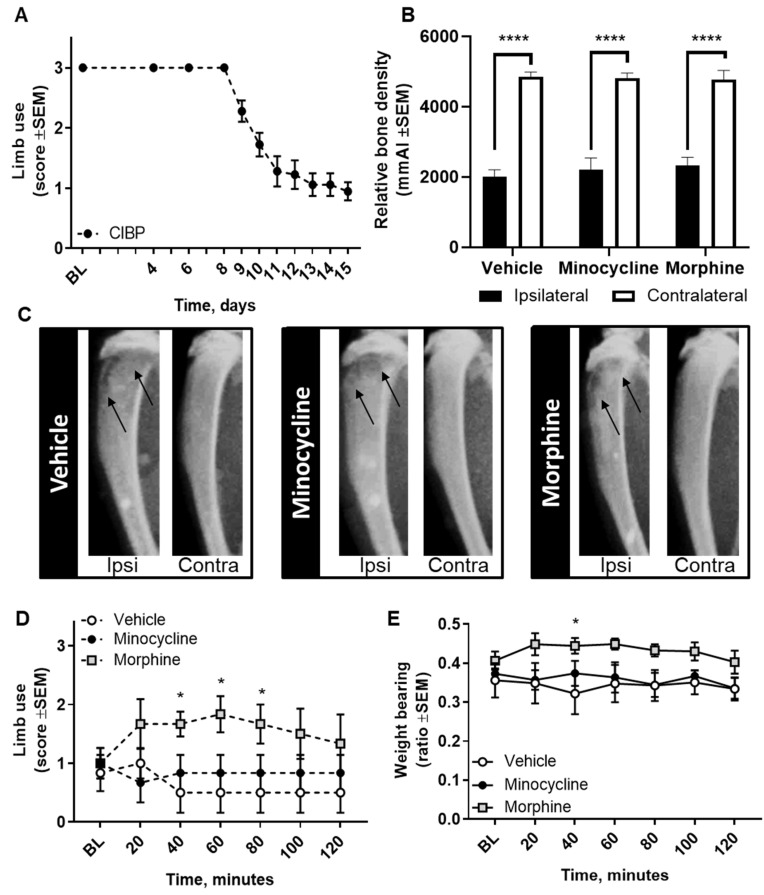
Pharmacological glial modulation in the CIBP model. (**A**) Cancer-bearing rats developed a pain-like phenotype from post-surgical day 8. Upon reaching a limb-use score of 1, rats receive an intrathecal (i.t.) injection of minocycline (100 µg) or morphine (10 µg) and their behavior was assessed at 20-min intervals during 2 h. (**B**) At euthanasia, the relative bone density of ipsilateral cancer-bearing tibias was significantly lower than the contralateral tibia in all experimental groups. (**C**) Representative images of the ipsilateral and contralateral tibias of cancer-bearing rats receiving a single intrathecal administration of vehicle, minocycline, or morphine. Arrows indicate areas of bone degradation. (**D**) Spinal administration of minocycline did not alter limb-use scores, while morphine administration induced anti-nociception 40 to 80 min after administration. (**E**) Similarly to the limb-use test, intrathecal minocycline did not alter the weight-bearing ratio, but spinal morphine administration caused a significant increase 40 min after administration and showed a positive trend 60 min after administration (*p* = 0.0574). (**B**–**E**) Vehicle-treated n = 6; minocycline-treated n = 6; morphine-treated n = 6. * *p* < 0.05; **** *p* < 0.0001. Data are presented as mean ± SEM. BL: baseline.

To further confirm that microglial reaction is not a main feature of the Walker 256 model of CIBP, we investigated whether pharmacological inhibition of spinal microglia had an anti-nociceptive effect. Female Sprague Dawley rats inoculated with Walker 256 cancer cells developed pain-related behaviors over time, seen as a pronounced decrease in limb-use scores from post-surgical day 8 (Figure 8A). Upon reaching a limb-use score of 1, rats were randomized to receive a single intrathecal dose of the microglial inhibitor minocycline (100 µg), morphine (10 µg), or vehicle. Intrathecal administration of 100 µg minocycline had no effect on the observed pain-related behavior as compared with vehicle (Figure 8C,D). In contrast, intrathecal administration of 10 µg morphine caused a significant amelioration of pain-related behaviors in cancer-bearing rats compared with vehicle, seen as a significant increase in the limb-use score and weight-bearing ratio (*F*_12,90_ = 2.73, *p* < 0.05 and *F*_12,90_ = 0.57, *p* < 0.05, respectively; n = 6; Figure 8C,D). Importantly, at euthanasia the relative bone density of the ipsilateral tibias did not differ between groups, indicating a similar extent of osteolytic lesions (*F*_2,30_ = 0.13, *p* > 0.05; n = 6; Figure 8B). Within groups, a significant difference was seen in the relative bone density comparing the ipsilateral and contralateral tibias (*F*_1,30_ = 202.20, *p* < 0.0001 ipsilateral vs. contralateral; n = 6; Figure 8B).

## 3. Discussion

Bone is the preferred metastatic site for prevalent cancers, such as breast and prostate cancer [33] and metastatic bone disease can be very painful and difficult to treat with the available analgesics [9]. While increasing efforts are being made to identify new analgesics for the treatment of chronic pain conditions, most promising targets lack translational value and fail in the early stages of clinical trials, suggesting that a better characterization of the animal models is needed [12,13]. In models of painful bone metastases, a recurrent debate is whether spinal microglia are involved in the transmission of CIBP, with contradictory data being reported [20,26,30,31,32,34,35,36]. Our results suggest that microgliosis is not a main feature of CIBP, independent of disease stage, cancer cell line, or sex of the rat. In accordance, acute intrathecal administration of the microglial inhibitor minocycline failed to attenuate pain-like behaviors in cancer-bearing female rats.

To better understand the role of spinal microglia in CIBP, we used the female Walker 256 model in which Walker 256 carcinoma cells were intratibially inoculated in syngeneic Sprague Dawley rats, a model of CIBP in which microgliosis is commonly described [26,30,31,32]. We applied immunohistochemistry to investigate the expression of Iba-1 and P-p38 MAPK, two markers used to detect microglial reaction [25], and saw no increase in the numbers of spinal Iba-1^+^ or P-p38^+^ cells in cancer-bearing rats. While our results are contradictory to many of the reported observations, they are in agreement with other published data [35,37].

The p38 is one of three major members of the MAPK family, the others being extracellular signal-regulated kinase (ERK) and c-Jun N-terminal kinase (JNK). The p38 is a global downstream signaling kinase present in many CNS cell types, but, interestingly, the phosphorylated version (P-p38) is in many pain models found to be primarily activated in microglia and barely found in neurons, astrocytes, or oligodendrocytes [38,39,40,41,42,43,44,45]. This was first observed in models of peripheral neuropathic pain [39,40,45] but has also been seen a various time points in cancer-induced bone pain [44], in the development of tolerance to morphine analgesia [41], and after intrathecal injection of lipopolysaccharide (LPS) [42]. In agreement with these findings, we observed that about 97% of P-p38+ cells were also positive for Iba-1 in the ipsilateral spinal cord dorsal horn of SNI-operated rats. Therefore, P-p38 was used as an additional marker of microglia reaction. The immunohistochemical analysis was performed at the lumbar spinal cord segmental level 4 (L4), as robust microglia reaction previously has been demonstrated in that area in the Walker 256 model of CIBP [26,37,44,46,47,48,49,50].

Complicating the debate on the role of microglia in the transmission of the nociceptive signal, temporal activation of microglia has been observed in several pain models, including models of CIPB [26,31,32,34], in which the findings are again contradictory. It has thus been reported that microglia are important for the initiation of CIBP, but not its maintenance [26,32], as opposite to that microglia are important for the maintenance of CIBP, but not the initiation [30] and also that microglia are involved in both phases [31]. To further explore this, we investigated microglial reaction in the early and late phases of CIBP, but found no significant increase in the numbers of Iba-1^+^ or P-p38^+^ cells just before the initiation of nociception (post-surgical day 6), as the nociceptive-phenotype commenced (post-surgical day 8), or at the progressed stage, where the humane endpoint was reached. Thus, we did not observe a temporal microglial reaction in the Walker 256 model of CIBP.

Sexual dimorphism in microglial reaction has also been described, and it has been suggested that pathological microglia signaling is fundamentally different between male and female rodents in models of peripheral neuropathic pain [27,29]. This is in contrast to CIBP, where microglial reaction is reported in both male and female cancer-bearing rodents [26,30,32,36,48,51]. In our study, we found no indication of microglial reaction in either female or male rats inoculated with Walker 256 cancer cells, again adding to the evidence that microglial reaction is not a uniform feature over the same models of CIBP.

The occurrence of microglial reaction is likely affected by the choice of cancer cell line, animal species, and strain. In this study, we inoculated male Sprague Dawley rats with the two syngeneic Walker 256 and MRMT-1 breast carcinoma cell lines and found no microglial reaction in either model. In a model of secondary brain tumors, Walker 256 cells obtained from two different cell banks (American Type Culture Collection (ATCC) and Cell Resource Centre for Medical Research at Tohoku University (CRTCU)) showed differences in microglial reaction, with the inoculation of the ATCC cell line inducing widespread, ill-defined labelling throughout the brain tumor mass and the CRTCU cell line showing sparse but specific, discrete labelling [52]. These results indicate that even the choice of cell line origin may influence the development of microgliosis. Moreover, both mouse and rat models of CIBP show contradictory findings on microglial reaction [20,26,30,31,32,34,35,36] and it has been reported, for models of neuropathic and inflammatory pain, that Sprague Dawley rats purchased from different vendors show profound differences in the nociceptive hypersensitivity and response to analgesics [53]. Together, these findings suggest that the cancer cell line, the animal, and most likely the interaction between the two can influence whether microglia play a role in chronic CIBP.

The identification of new targets guides drug discovery, and it is crucial that our preclinical models have high translational validity [54]. The animal models of CIBP are generally considered appropriate, as good face and predictive validity have been demonstrated, i.e., the animal models present with tumor burden, bone resorption or formation, and pain-like behaviors that can be attenuated with drugs known to alleviate pain in patients, such as morphine [55,56]. In the Walker 256 model of CIBP, we observed the expected anti-nociceptive effect following acute spinal administration of morphine. To modulate microglia pharmacologically, we evaluated the effect of intrathecal administration of minocycline in cancer-bearing rats. In agreement with the lack of microglial reaction, minocycline did not cause any alteration of the pain-like phenotype of cancer-bearing rats. While single intrathecal administration of minocycline has previously been reported as anti-nociceptive in this model [30,46,57], repeated dosing may be necessary to attenuate CIBP. There is only little evidence of the involvement of microglia in human pain states and, so far, most clinical trials of microglia modulatory agents have not had robust or consistent results [58,59,60,61]. A trial using minocycline suggested that perioperative administration decreased persistent pain after lumbar discectomy in a subgroup of patients presenting with deep spontaneous pain at baseline [58], and a small effect was found in a study of lumbar radicular neuropathic pain [59]. The lack of more pronounced effects may be due to the heterogeneity of the patient population, which may be reflected in the animal models.

Taken together, our preclinical data suggest that microglial reaction is not a main feature of CIBP. Additional evaluation of spinal cytokine levels, protein expression, or RNA analyses of spinal tissue could help further elucidate this discrepancy between models of CIBP. However, as reports on the role of microglial in the transmission of CIBP are conflicting, it could be speculated that microglia-targeting treatments are beneficial for a subset of patients with painful metastatic bone disease; however, this would need to be confirmed in the clinical setting.

## 4. Materials and Methods

### 4.1. Cell Culture

Walker 256 cells (Rikken Cell Bank, Ibaraki, Japan) were cultured in Roswell Park Memorial Institute (RPMI) medium without phenol red, supplemented with 10% Fetal Bovine Serum (FBS) and 1% penicillin-streptomycin-glutamine. Cells were routinely cultured at 37 °C and 5% CO_2_ for at least 14 days before surgical inoculation. On the day of surgery, cells were harvested with 0.05–0.1% trypsin-EDTA (Gibco, Life Technologies, Paisley, UK) and resuspended in phosphate buffered saline (PBS) or Hank’s Balanced Salt Solution (HBSS). MRMT-1 cells (Rikken Cell Bank, Ibaraki, Japan), transfected with *Luc2* as previously described [62], were cultured in the same fashion. Unless otherwise specified, all cell reagents were purchased from Invitrogen, Nærum, Denmark.

### 4.2. Animals

Five- to 12-week-old male and female Sprague Dawley rats (Taconic Biosciences, Tornbjerg, Denmark) were housed in groups of four or five in standard, individually ventilated cages (Naxgen Rat 1800, floor area: 1805 cm^2^, Herfølge, Denmark) in a specific pathogen-free facility at a 12-h light/dark cycle (light on at 7:00 a.m.), with normal bedding (Tapvei 2HV, Tapvei, Estonia) and ad libitum access to water and food (Altromin 1314, Brogaarden, Denmark). Environmental enrichment was provided as red translucent shelters, gnawing sticks, and nesting material (Tapvei, Estonia). Animal experiments were approved by the Danish Animal Experiments Inspectorate (Copenhagen, Denmark), ethical code: 2014-15-0201-00031, and complied with the Danish Act on Animal Experiments (LBK No. 474 of 15/05/2014) and with the Animal Research: Reporting of In Vivo Experiments (ARRIVE) guidelines for reporting animal experimentation. All efforts were made to minimize animal suffering. All animals were used in a single experiment and were drug/test naïve prior to the initiation of the experiment. Welfare assessments (including observation of weight, presence of porphyrin, fur condition, food/water intake, stools, and development of abnormal behaviors) were conducted regularly throughout the experimental time. A total of 87 rats were included in the cancer experiments and 25 in the SNI studies. For the cancer studies, 28 rats were excluded due to lack of cancer development (n = 26), growth of extraosseous tumor (n = 1), or bad perfusions (n = 1). SNI-operated rats that failed to show mechanical hyperalgesia (n = 1) or sham-operated rats showing hyperalgesia (n = 2), as measured in the von Frey test, were excluded from the study.

### 4.3. CIBP Surgery

CIBP surgeries were conducted under isoflurane anesthesia (3–4% for induction, 2–2.5% for maintenance; Nomeco or Baxter, Søborg, Denmark). Carprofen (5 mg/kg, subcutaneous (s.c.), Norodyl, Pfizer, Denmark) was administered before surgery for post-surgical analgesia. Intratibial inoculation of cancer cells was conducted, as previously described [17,63]. The medial distal side of the right hind limb was shaved and disinfected with 70% ethanol and an incision of approximately 1 cm was pierced in the skin with a scalpel. The tibia was exposed and a 0.7-mm dental drill was used to drill a hole toward the proximal intramedullary cavity of the tibia, in which a catheter (Smiths Medical, Ashford, UK) was inserted and 10 µL of the cancer cell suspension or vehicle was inoculated. Walker 256 cells were injected in a concentration of 10^5^ cells/10 µL except for the comparison study (comparing MRMT-1-inoculated rats and Walker 256-inoculated rats), in which both MRMT-1 and Walker 256 cells were injected in a concentration of 5 × 10^3^ cells/10 µL. The hole was closed with restorative cement (IRM cement, Dentsply, Denmark), the wound profusely irrigated with saline, and the skin sealed with two metal clips (11 × 2.5 cm, Agnthos, Sweden). Xylocaine gel (2% *w*/*v*; AstraZeneca, Copenhagen, Denmark) was applied on the surgical site. Clips were removed approximately 4–7 days post-surgery. Animals were stratified into cancer-bearing or sham groups according to weight.

### 4.4. Spared Nerve Injury (SNI) Surgery

SNI surgeries were conducted under isoflurane (3–4% for induction, 2–2.5% for maintenance; Baxter A/S, Søborg, Denmark) or a 1:1 Hypnorm:Dormicum cocktail (fentanyl 0.630 mg/kg fluanisone 20 mg/kg and midazolam 10 mg/kg, VetaPharma, Hvidovre, Denmark and Accord, Denmark) in sterile water and injected s.c. Carprofen (5 mg/kg, s.c., Norodyl, Pfizer, Denmark) was administered before surgery and 24 and 48 h after to control post-surgical pain. Upon anesthesia, the posterior side of the right hind limb was shaved and disinfected with 70% ethanol. Using a scalpel, an incision of approximately 1 cm was pierced in the skin and a scissor was used to make a separation of the biceps femoris muscle, exposing the three branches of the sciatic nerve. Ensuring that the sural nerve branch was untouched, the tibial and peritoneal nerves were tightly ligated with 4.0 non-absorbable silk suture (Perma Hand, Ethicon, Brøndby, Denmark) and 2–3 mm of the nerve excised to prevent nerve regeneration. Sham animals underwent a similar procedure without ligation or excision of any nerve branches. Muscle and skin were then sutured (3-0 Vicryl polyglactin, absorbable thread, Ethicon, Brøndby, Denmark) and glued (Vetbond, 3M, Kruuse, Langeskov, Denmark), and xylocaine gel (2% *w*/*v*; AstraZeneca, Copenhagen, Denmark) was applied to the surgical wound. Animals were stratified according to baseline paw withdrawal threshold into sham or SNI.

### 4.5. Drug Interventions

Minocycline (100 µg, HelloBio, Bristol, UK) and morphine (10 µg, Takeda Pharma A/S, Taastrup, Denmark) were prepared fresh on the day of injection; osmolarity and pH were measured and adjusted to 294 osmol/kg and pH 7, respectively. Saline was used as vehicle. Rats were randomized to receive drug or vehicle. Drugs were administered by a different researcher than the one conducting behavioral testing to ensure complete blinding throughout the experiment. All drugs were intrathecally administered through lumbar puncture in a volume of 10 µL. Briefly, rats were anesthetized with isoflurane (4–5% for induction, 1.5–2% for maintenance, Baxter, Denmark) and the back was shaved and disinfected. A 23 gauge needle attached to a Hamilton syringe was inserted at the level of the lumbar segment L5–L6. Needle placement was confirmed by flinching of a hind limb or tail. One rat showed signs of motor impairment following intrathecal injection that lasted about 60 min; this was probably due to spinal damage during lumbar puncture and the animal was excluded from analyses. No other adverse effects were observed in any of the other experimental groups.

### 4.6. Behavioral Tests

All behavioral testing was conducted during the light phase (7:00 a.m. to 7:00 p.m.). Within each experiment, behavioral tests were performed by the same researcher, blinded to experimental groups and treatment.

#### 4.6.1. Limb Use

The limb-use test was used to assess the gait of cancer-bearing and sham animals. Rats were placed in groups in a transparent plastic cage (650 × 250 × 200 mm) and left to acclimatize for 10 min. Thereafter, rats were individually observed for three minutes and scored as follows: 3 = normal gait, 2 = insignificant limping, 1 = significant limping and shift in bodyweight distribution toward the healthy limb, 0 = lack of use of ipsilateral limb. A limb-use score of 0 was set as humane endpoint.

#### 4.6.2. Weight Bearing

The weight-bearing test was used to assess the bodyweight distribution of cancer-bearing and sham animals. Rats were individually placed in an Incapacitance Tester (MJS Technology Ltd., Buntingford, Hertfordshire, UK) consisting of two separate scales measuring the individual load on each hind limb. The amount of weight placed on the hind limbs for four seconds was measured three times for each animal. The weight-bearing ratio was calculated as average weight placed on the ipsilateral limb divided by the total average weight placed on both hind limbs.

#### 4.6.3. Von Frey

Mechanical hypersensitivity of SNI-operated and sham animals was measured with von Frey monofilaments (North Coast Touch Test, CA, USA) following the Dixon up-down method [64]. Briefly, rats were placed in individual cages on an elevated mesh floor and allowed to acclimatize for at least 30 min. Rats were not tested while grooming or sleeping. Starting at 2.0 g, von Frey monofilaments (0.40–15.0 g) were sequentially applied to the right outer plantar area of the ipsilateral hind limb for 2–3 s. A positive response was followed by the application of a weaker filament, while a negative response was followed by the application of a stronger filament. Positive responses were defined as withdrawal, stamping, or shaking the paw in three out of five applications of the same monofilament. The resulting pattern of positive and negative responses was used to calculate the 50% Paw Withdrawal Threshold (PWT) as defined by Dixon [64].

### 4.7. X-ray Imaging

The relative bone density of the ipsilateral tibia of cancer-bearing and sham rats was evaluated through X-ray imaging, as previously described [62]. Briefly, rats were anesthetized with isoflurane (3–4% for induction, 2–3% for maintenance, Nomeco or Baxter, Denmark) and placed in a Lumina XR Apparatus (Caliper Life Science, Teralgene, Belgium). X-ray images of the tibia were captured and calibrated to a standard aluminum wedge. Image analyses was conducted in Image J (National Institute of Health, Madison, Wisconsin, USA) by measuring the grayscale value of the distal tibial area and subtracting the mean grayscale value of two soft-tissue regions; the final value was then normalized against the standard aluminum wedge. X-ray images were analyzed by a researcher blinded to the experimental groups and treatment.

### 4.8. Tissue Extraction

Rats were deeply anesthetized through s.c. administration of a 3:4 Ketaminol/Dexdormitor cocktail (Ketaminol, MSD Animal Health, Copenhagen, Denmark; Dexdormitor, Orion Pharma, Copenhagen, Denmark; s.c. injection of 1.75 mL/kg) or a 1:1 Hypnorm:Dormicum cocktail (fentanyl 0.630 mg/kg, fluanisone 20 mg/kg, and midazolam 10 mg/kg, VetaPharma, Hvidovre, Denmark and Accord, Denmark) in sterile water and injected s.c. The abdominal cavity was cut open, the diaphragm punctured, and the thoracic cage cut to expose the heart. A 23-G needle connected to a MasterFleex L/S pump (Buch & Holm, Herlev, Denmark) was inserted into the left ventricle of the heart and the right atrium was cut open. Following PBS perfusion, a flow of ice-cold 4% paraformaldehyde (PFA) with 0.12% picric acid (VWR, Leicestershire, UK) was applied. The lumbar region of the spinal cord was extracted and post-fixated overnight in 4% PFA with 0.12% picric acid at 4 °C. Tissue was dehydrated in 30% sucrose at 4 °C, embedded in Tissue-Tek O.C.T. compound (Sakura, Japan), and stored at −80 °C until further analyses.

### 4.9. Immunohistochemistry

L3–L4 spinal cord segments were serially sectioned on a cryostat (Leica CM 3050 S; Leica Microsystems A/S, Herlev, Denmark) and thaw mounted onto SuperFrost plus microscope slides (Menzel Glazer; VWR, Denmark). Sections were 30 µm thick and collected at a distance of 240 µm. Sections were washed 3 × 5 min in phosphate-buffered saline (PBS) and 1 × 5 min in 0.1% Triton X-100 in PBS (T-PBS) to remove Tissue-Tek O.C.T. compound. For double immunostaining, sections were blocked for 1 h in 5% donkey serum (Jackson ImmunoResearch, Cambridgeshire, UK) in T-PBS at room temperature (RT), washed 3 × 5 min in T-PBS, and incubated overnight at 4 °C with rabbit phospho-p38 MAPK (Thr180/Tyr182) monoclonal antibody (1:500, Bionordika, Cell Signaling Technology 4511S, Herlev, Denmark). The following day sections were washed 3 × 5 min in PBS and incubated overnight at 4 °C with goat anti-Iba-1 polyclonal antibody (1:1000, Abcam ab107159, Cambridge, UK). On the third day, sections were washed 3 × 5 min in PBS, incubated two hours at RT with donkey anti-rabbit Alexa Flour 594 (1:300, Fisher Scientific 10798994, Slangerup, Denmark), washed 4 × 5 min in PBS, incubated two hours at RT with donkey anti-goat Alexa Flour 488 (1:1000, Fisher Scientific 10246392, Slangerup, Denmark), washed 3 × 5 min with PBS, and counterstained with DAPI (1:30,000, FluoroPure, Thermo Scientific, Eugene, OR, USA). After a final 3 × 5 min wash in PBS, sections were mounted with Dako fluorescence mounting medium (Agilent Technologies Denmark ApS, Glostrup, Denmark) and cover slipped. Single immunostaining for Iba-1 was performed similarly using 1% goat-serum (Jackson ImmunoResearch Europe Ltd., Cambridgeshire, UK) in T-PBS, rabbit anti-Iba-1 antibody (1:1000, Wako Pure Chemical Industries, Ltd., Neuss, Germany), and goat anti-rabbit Alexa Flour 594 (1:000, Thermo Fisher Scientific A-11012, Slangerup, Denmark). Unspecific staining of secondary antibodies and background fluorescence were investigated by the omission of primary or secondary antibodies. No staining for Iba-1 or P-p38 was observed.

### 4.10. Image Analyses

Images were acquired using a Zeiss Axioskop2 microscope (Zeiss, Feldbach, Switzerland) equipped with an Axiocam MRm camera (HAL100) (Zeiss, Feldbach, Switzerland) and a fluorescent Transmitter (HXP 120, Digital Scientific, Cambridge, UK). Images from single and double immunostainings were acquired with 20× and 40× objectives, respectively. Image analysis was performed in Image J (Wayne Rasband; National Institutes of Health, Bethesda, MD, USA) using image overlay. Numbers of Iba-1 positive (Iba-1^+^) and P-p38^+^ cells were for double immunostaining quantified in a predetermined area of 347 × 260 µm^2^, placed in the medial part of the gray matter, and aligned with the dorsal edge of the gray matter to include parts of laminae I–IV of the ipsilateral or contralateral dorsal horn (Figure 2A). For single immunostaining, the total gray matter covering laminae I-IV was outlined and the numbers of Iba-1^+^ cells quantified. A cell was only counted if it displayed an obvious nucleus, as judged by DAPI stain. From each rat, three of the collected sections were randomly chosen to cover a longitudinal distance of minimum 580 µm and maximum 1200 µm, the numbers of Iba-1^+^ and P-p38^+^ cells counted, and the average number of cells included in the statistical analysis. The results are presented as cells per an area of 347 × 260 µm^2^.

Point scanning confocal microscopy was performed with a Leica SP5-X MP system (Leica Microsystems, Heidelberg, Germany) equipped with a 405-nm diode laser and a fast beamsplitter for excitation of DAPI, a white light laser for excitation of Alexa fluorophores, and a tunable acousto-optical beamsplitter. A sensitive hybrid pixel detector (HyD) was used for Alexa Flour 488, and standard photon multiplier tubes were used for the other laser lines. Images were acquired with LAS AF software (Leica) using a 40× objective with 1.3 magnification oil immersion. For determination of co-localization of P-p38^+^ cells with Iba-1^+^ cells, cells were quantified in a predetermined area of 450 × 338 µm^2^, aligned with the medial and dorsal edge of the ipsilateral dorsal horn gray matter covering most parts of laminae I–IV. Three SNI-operated rats were included in the analysis and a single L4 section counted from each rat. Cells that were positive for both P-p38 and DAPI were included as P-p38^+^, while cells that were positive for P-p38, DAPI, and Iba1 were included as co-localized with microglia.

### 4.11. Statistical Analyses

All data were analyzed in GraphPad Prism 8.0 (GraphPad Software, San Diego, CA, USA) or SAS (SAS Institute Inc., Cary, NC, USA) and all graphs were plotted in GraphPad Prism 8.0. Power calculations were performed in G*Power v3.1.9.7 [65]. Effect size was estimated on the basis of the number of Iba-1 cells in the ipsilateral dorsal horn of sham and SNI-operated rats. Considering effect size *d* = 4.22, α = 0.05, and a power of 0.95, the estimated sample size was n = 3. All immunohistological data and parametric behavioral data were analyzed by two-way ANOVA (repeated measures for behavioral data) followed by Sidak’s post hoc test for multiple comparisons. Nonparametric behavioral data were analyzed by Friedman’s two-way test and Wilcoxon’s two-sample test for single time points. In the pharmacology study, nonparametric data were analyzed by one-way ANOVA followed by Dunnett’s test for multiple comparisons at single time points. Single animals were defined as experimental units, and sample size was empirically determined from pilot studies. All data are presented as mean ± SEM.

### 4.12. Data Sharing Statement

All data and protocols generated during the studies described in this manuscript are freely available upon reasonable request.

## 5. Conclusions

Here we demonstrated that microglial reaction is not a main feature of either the Walker 256 or MRMT-1 model of CIBP, independent of disease stage or sex of the rat. In accordance, minocycline failed to attenuate pain-like behaviors in cancer-bearing male rats. This finding adds to the debate that even within the same models of CIBP significant variations are seen in disease features that are considered possible drug targets. However, instead of dismissing the translational validity of the models, we suggest that these diverse features may actually reflect the heterogeneity of the patient population. Thus, further characterization of the models is crucial to understand, and perhaps increase, their translational validity.

## Supplementary Materials

The following are available online at https://www.mdpi.com/2072-6694/12/10/2740/s1, Figure S1: Development of nociception and osteolytic bone degradation in Sprague Dawley rats inoculated with Walker 256 or MRMT-1 cancer cells; Figure S2: Development of nociception and osteolytic bone degradation in male Sprague Dawley rats following Walker 256 cancer cell inoculation.
