# Supplementary material for: Modulation of Rat Cancer-Induced Bone Pain is Independent of Spinal Microglia Activity
Source: Cancers (Basel). 2020 Sep 24;12(10):2740. doi: 10.3390/cancers12102740 (PMC7598664; doi:10.3390/cancers12102740)
Supplement: Supplementary file 1 [file cancers-12-02740-s001.pdf]

cancers

Supplementary Figures

Modulation of Rat Cancer-Induced Bone Pain Is Independent of Spinal Microglia Activity

Marta Diaz-delCastillo, Rie Bager Hansen, Camilla K. Appel, Lykke Nielsen, Sascha N. Nielsen, Konstantinos Karyniotakis, Louise M. Dahl, Rikke B. Andreasen and Anne-Marie Heegaard

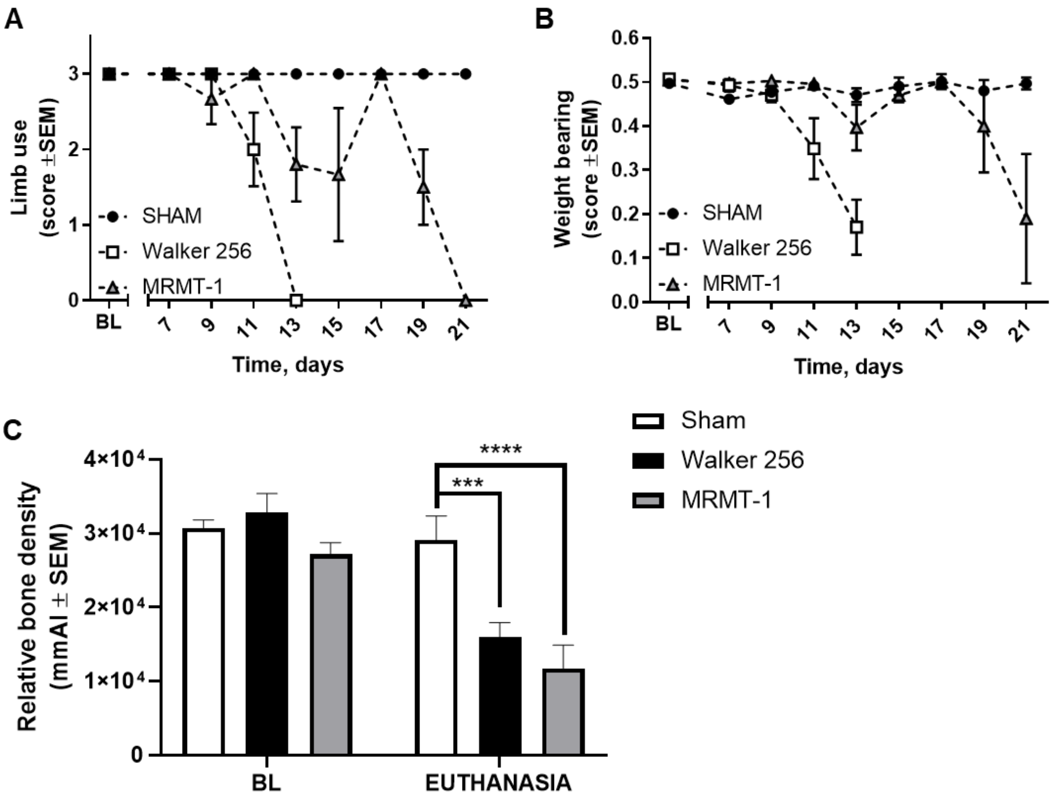

Figure S1. Intrafemoral innoculation of Walker 256 or MRMT-1 cells induces the decrease of limb use scores (**A**) and weight bearing ratios (**B**) in cancer-bearing rats, but not in sham. (**C**) Both MRMT-1 -innoculated tibias and Walker 256 -inoculated tibias show a significant decrease in relative bone density at euthanasia, compared with sham. Data are presented as mean ± SEM. Sham n = 10; Walker 256 n = 7; MRMT-1 n = 6. *** $p < 0.001$; **** $p < 0.0001$.

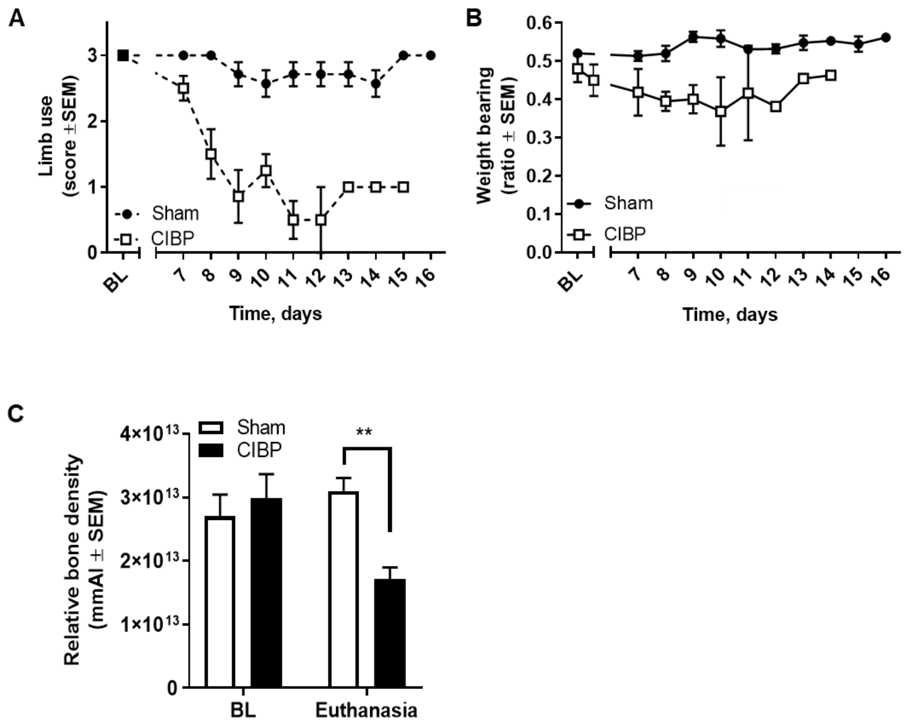

Figure S2. Male Sprague Dawley rats show a decrease in limb use scores (**A**) and weight bearing ratios (**B**) following intratibial inoculation of Walker 256 carcinoma cells. (**C**) Cancer-bearing tibias show a significant decrease in relative bone density at euthanasia, compared with sham. Data are presented as mean ± SEM. Sham n = 7; CIBP n = 8. ** $p < 0.01$; **** $p < 0.0001$.
